# Disparities in Connectivity Profiles Between Structural and Functional Networks in Patients With Temporal Lobe Epilepsy

**DOI:** 10.1002/brb3.70935

**Published:** 2025-09-27

**Authors:** Yu Zhou, Liangchao Du, Fangfang Xie, Dingyang Liu, Zhuanyi Yang, Chao Deng, Chunyao Zhou, Zhiquan Yang

**Affiliations:** ^1^ Department of Neurosurgery Xiangya Hospital Central South University Changsha Hunan Province China; ^2^ Department of Radiology Xiangya Hospital Central South University Changsha Hunan Province China; ^3^ Department of Orthopaedics Xiangya Hospital Central South University Changsha Hunan Province China

**Keywords:** duration of epilepsy, functional connectivity, structural connectivity, temporal lobe epilepsy

## Abstract

Temporal lobe epilepsy (TLE) is one of the most prevalent forms of epilepsy. Recent advancements in research have increasingly categorized TLE as a network disorder, focusing on its network characteristics. Prior studies predominantly examined changes in connectivity properties within a single network, with limited attention to the distinctions between different connectivity networks. Both structural and functional connectivity serve as quantitative measures of connection strength among network nodes, yet they differ significantly. Functional connectivity (FC) highlights short‐term cooperation among nodes, whereas structural connectivity (SC) underscores long‐term cumulative effects of structural alterations. In this study, we constructed a comprehensive spectrum of structural and functional network connectivity in TLE patients and investigated the modulatory impact of seizure duration on both types of connectivity. Our findings indicate that the functional and structural connectivity of the brain displays inconsistent coupling patterns and heterogeneity. Specifically, we observed pervasive reductions in both structural and functional connectivity. As seizure duration increased, SC decreased, particularly in regions adjacent to the lesion. Additionally, there was a general decline in FC. However, we noted a significant increase in abnormal FC between nodes of the default mode network near the midline as seizure duration prolonged. These results suggest that the evolutionary patterns of structural and functional connectivity may differ in patients with TLE, indicating potential network reorganization.

## Graphical abstract

Structural and functional network connectivity characteristics in TLE and their correlation with seizure duration

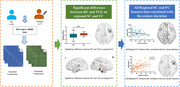

**Abbreviations**
DMN: default mode network
DTI: diffusion tensor imaging
FC: functional connectivity
MRI: magnetic resonance imaging
rs‐fMRI: resting state magnetic resonance imaging
SC: structural connectivity
SEEG: stereoelectroencephalography
TLE: temporal lobe epilepsy

## 1 Introduction

Recently, our understanding of TLE has expanded beyond structural lesions to include network pathologies. While initial studies primarily focused on structural lesions in the hippocampus and medial temporal lobe, researchers now acknowledge the significance of network mechanisms in seizure onset, propagation, and cognitive impairment (DeSalvo et al. 2014; Li et al. 2022; Morgan et al. 2020; Wan et al. 2023). Consequently, advanced multimodal neuroimaging techniques, such as DTI (diffusion tensor imaging) and rs‐fMRI (resting state magnetic resonance imaging), are favored for constructing brain network connectomics to elucidate the intricate relationship between brain functional and structural networks (Kelly et al. 2012; Smith et al. 2013).

Generally, SC focuses on the physical structure of the system and how it is connected, whereas FC emphasizes the interaction and information transfer capabilities between components. Two studies revealed that the extent of SC and FC reduction was inconsistent across the same network nodes in patients with TLE (Chiang et al. 2015; Trimmel et al. 2021), illustrating that spatial heterogeneity exists in their patterns of change. The FC group demonstrated a wider range of decreases across cortical and subcortical nodes than the SC group, especially in the nodes of the default mode network (DMN) (Liu et al. 2016; Voets et al. 2012), suggesting differences in the recombination patterns of structural and functional networks.

Although some studies have revealed the temporal evolution of brain functional network topology in patients with TLE and highlighted the dynamic nature of brain plasticity (Liang et al. 2021; Morgan et al. 2015). However, there is still controversy about the reorganization pattern of brain structure and function in patients with TLE, especially whether it is related to seizure duration. Previous studies have described dynamic changes in the FC and SC in TLE under pathological conditions. For example, postsurgical remodeling of contralateral language networks has been demonstrated in patients with TLE, suggesting enhanced FC (Foesleitner et al. 2021). Some researchers have found that the FC–SC coupling index in the right inferior frontal gyrus increases with the duration of seizures in TLE, which may indicate a compensatory process in the attention network (Chiang et al. 2015). Furthermore, another study revealed that longer seizure duration was associated with reduced FC from the ipsilateral to the contralateral temporal, precuneus, and mid‐cingulate gyri (Chiang et al. 2016). Alterations in connectivity patterns within neural network nodes may serve as compensatory mechanisms for disrupted connectivity in other regions or to accommodate epileptic loads.

However, our current understanding of the reorganization pattern of structural and functional connectivity in patients with TLE is still incomplete, and further studies are needed. In this study, in addition to evaluating alterations in structural and functional connectivity in TLE patients compared with healthy controls (HC), we also assessed the correlation between disease duration and structural and functional connectivity. By exploring the network topological properties and their correlation with seizure duration, we can enrich insights into neural network plasticity to understand the underlying mechanisms and potentially develop innovative approaches for diagnosing, treating, and intervening in neurological diseases.

## 2 Materials and Methods

### 2.1 Patients

This study included a total of 71 patients who underwent unilateral TLE resection surgery and received preoperative assessment at the Functional Neurosurgery Department of Xiangya Hospital, Central South University, China. The cohort comprised 32 males and 39 females, with a mean age of 31.0 ± 11.5 years. Inclusion criteria were: (1) diagnosis of drug‐resistant epilepsy and (2) absence of contraindications for surgical resection. Exclusion criteria encompassed progressive neurological disorders, lesions beyond the temporal region, and severe mental disorders. The lateralization of the epileptic focus was determined based on the patient's symptomatology, imaging findings, and electroencephalography. Additionally, a group of 48 individuals who had no history of epilepsy or any other chronic neurological or psychiatric disorders was selected as HC. Detailed demographic information is presented in Table 1. The present study obtained approval from the Institutional Review Board and Ethics Committee of Xiangya Hospital, in accordance with the principles set forth in the Helsinki Declaration. Written informed consent was obtained from all participants.

**TABLE 1:** Clinical and demographic characteristics of the participants.

	HC	Patients	*p* Value
No. of participants, *n*	48	71	—
Age, years, mean ± SD	30.4 ± 8.4	31.0 ± 11.5	0.747
Years of education, years, mean ± SD	11.7 ± 2.7	11.4 ± 2.9	0.668
Sex (M/F)	30/18	32/39	0.062
Age at seizure onset, years, mean ± SD	—	16.7 ± 12.3	—
Years of seizure duration, years, mean ± SD	—	14.6 ± 9.4	—
TLE laterality, L/R	—	35/36	—
Histological type, HS/other	—	57/14	—
Seizure type, FTBTC/focal	—	56/15	—

Abbreviations: FTBTC, focal to bilateral tonic–clonic; HC, healthy control; HS, hippocampal sclerosis.

### 2.2 Magnetic Resonance Imaging Acquisition

rs‐fMRI data from all participants were acquired using a 3.0 Tesla Siemens Prisma magnetic resonance imaging (MRI) system equipped with a standard 32‐channel head coil. rs‐fMRIs were obtained using an echo‐planar imaging sequence. The specific acquisition parameters were as follows: repetition time = 720 ms, echo time = 37 ms, acquiring 64 axial slices with a thickness and gap of 2.5 mm each, flip angle = 52°, matrix size = 90 × 90, field of view = 225 mm × 255 mm, resulting in voxel size of 2.5 mm × 2.5 mm × 2.5 mm per volume scan lasting for approximately 9 min and 45 s.

DTI data were acquired using a multi‐shell EPI sequence with the following specifications: TE = 72 ms, TR = 5400 ms, resolution = 2.0 × 2.0 × 2.0 mm^3^, flip angle = 90°, acquiring 75 axial slices with voxel size of 1.6 × 1.6 × 1.6 mm^3^, FOV = 215 × 215 mm^2^, *b* values ranging from 0 to 3000 s/mm^2^ in steps of 1000 s/mm^2^, and 96 diffusion directions. The EPI factor was set to 154.

### 2.3 Data Processing

#### 2.3.1 SC Network Construction

First, the DTI data were preprocessed using FSL (https://fsl.fmrib.ox.ac.uk/fsl/fslwiki), following a series of steps: (1) conversion of the data format from the Digital Imaging and Communications in Medicine to the Neuroimaging Informatics Technology Initiative format. (2) Extraction of the b0 image and subsequent noise removal. (3) Application of the Brain Extraction Tool (BET) for skull stripping and obtaining brain masks. (4) Implementation of eddy current correction. (5) Computation of fundamental diffusion metrics such as Fractional Anisotropy (FA) and Mean Diffusivity. After preprocessing, the data were transferred to Mrtrix3 (https://www.mrtrix.org). To estimate the orientation distribution function, a multi‐shell, multi‐tissue‐constrained spherical deconvolution model was computed. Probabilistic fiber tracking was performed using the iFOD2 algorithm. We also computed streamlined weights to mitigate known biases in the tractography data using SIFT2. A 246 × 246 SC (two‐dimensional connectivity matrix based on degree centrality) matrix was constructed by tracing every possible pair of regions in the BrainNetome atlas, and specific structural networks were assigned to each subregion based on the Yeo‐7 parcellation map (Yeo et al. 2011). These structural networks encompass seven categories: the DMN, attentional networks, sensorimotor networks, visual networks, dorsal and ventral attention networks, and frontoparietal and subcortical networks. Considering the significant association of subcortical structures with TLE that has been reported in numerous studies, it is imperative to incorporate an eighth network, the subcortical network, as the Yeo functional map does not encompass these structures. Moreover, to standardize the lateralization of the epileptogenic focus, all connectivities of the left‐sided TLE patients were flipped to the opposite side (i.e., from left to right), resulting in all nodes and modules being labeled as either “ipsilateral” or “contralateral.”

#### 2.3.2 FC Network Construction

The rs‐fMRI data were preprocessed using the GRETNA toolbox (GRETNA; https://github.com/sandywang/GRETNA) based on SPM 12 (J. Wang et al. 2015), according to the following steps: (1) conversion of the DICOM format to NIfTI. (2) Exclusion of the initial 18 volumes to allow sufficient time for subjects to reach a resting state. (3) Correction of the slice timing. (4) Implementation of motion correction using a spatial realignment algorithm with 12 degrees of freedom. (5) Spatial normalization of data to the Montreal Neurological Institute (MNI) EPI template. (6) Application of spatial smoothing techniques. (7) Temporary removal of data trends. (8) Regression analysis performed on the global signal.

Subsequently, Pearson's correlation was employed to compute the average time series between each pair of brain regions from the Brainnetome Atlas (BN, http://www.brainnetome.org/, accessed on March 2, 2020) for each participant, resulting in an FC matrix (two‐dimensional connectivity matrix based on degree centrality) with dimensions of 246 × 246. Fisher's *Z*‐transformation was applied to the FC matrix to achieve a Gaussian distribution. A sparsity threshold of 10% was then implemented on the FC matrix, similar to the SC matrix; the FC matrix is also divided into eight modules. A comprehensive list of all the nodes and their respective networks is provided in Table S3.

### 2.4 Statistical Analysis

The data were subjected to statistical analyses using IBM SPSS Statistics version 25 (https://www.ibm.com/cn‐zh/spss). Independent sample *t*‐tests and one‐way analysis of variance (ANOVA) were used to compare intergroup differences in clinical characteristics between patients with TLE and HC. Functional and structural two‐dimensional connectivity matrices were constructed based on degree centrality. The correlation between seizure duration and SC and FC was assessed using partial correlation analysis, controlling for age, sex, and educational level as covariates. Nonparametric *t*‐tests were employed to compare the differences in whole‐brain network nodes SC and FC between individuals with TLE and HC; false discovery rate correction was used to control the false discovery rate. To control for the potential confounding effects of age, education level, and sex, we set these factors as covariates, which were adjusted to assess the relationship between SC and FC with seizure duration.

## 3 Results

### 3.1 Demographic and Clinical Characteristics

Table 1 displays the demographic characteristics of the study population. This study included 71 patients diagnosed with drug‐resistant TLE, with a mean age of onset at 16.7** ± **12.3 years and a mean preoperative seizure duration of 14.6** ± **9.4 years. A cohort of 48 age‐ and gender‐matched HCs was included in the study sample. No significant differences were found between the two groups in terms of sex distribution, age composition, or educational background (*p* > 0.05; Table 1).

### 3.2 Spatial Consistency of Regional SC and Its Relationship With Seizure Duration

**FIGURE 1:**
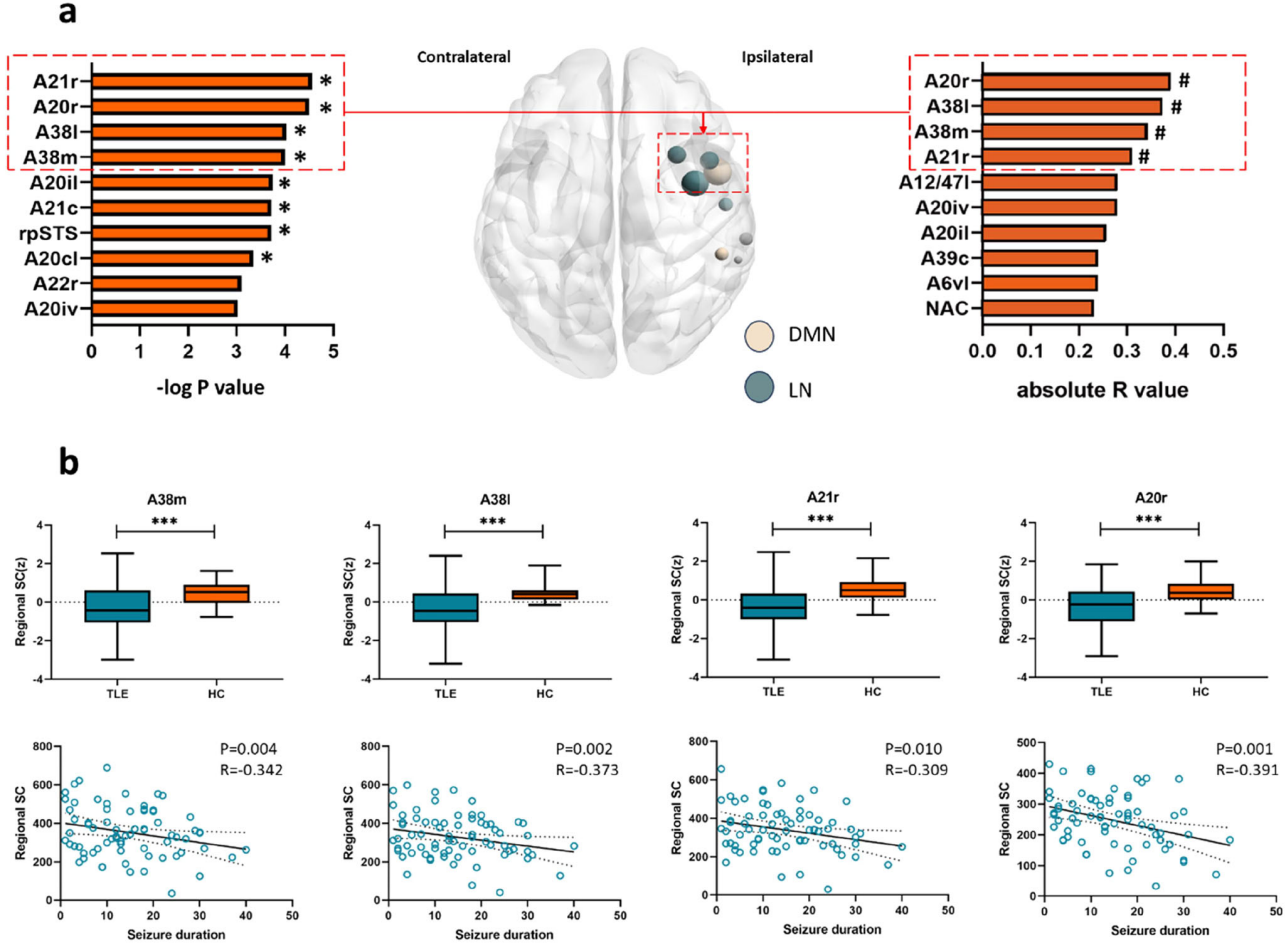
Clustering analysis was conducted to examine the differential SC between the group of patients with TLE and the HC groups, as well as perform correlation analysis between SC in the patient group and duration of epilepsy, considering all brain nodes. (a) The four nodes exhibiting the most significant decrease in regional SC were also found to have the strongest correlation between regional SC and seizure duration (A21r, A20r, A38l, and A38m; A21r is located in the default mode network, while A20r, A38l, and A38m are located in the limbic network). These nodes are located in the anterior temporal lobe, near the temporal pole. (b) For the aforementioned four nodes, SC between the patient group and the HC group was compared using unpaired *t*‐tests (*p < 0.05, **p < 0.01, ***p < 0.001), revealing a significant reduction in SC in the patient group compared to the HC group. (c) Partial correlation analysis revealed a significant negative correlation between the SC values of these four nodes and seizure duration (*p *< 0.05), with the line representing a linear regression plot for visual demonstration.

Compared with the HC group, the patient group exhibited significant reductions in SC within brain regions primarily associated with the default mode, frontoparietal network, and salience network. The significantly decreased nodes were distributed in the superior temporal gyrus (STG) (A38l/A38m), middle temporal gyrus (A21r/A21c), inferior temporal gyrus (A20r/A20il/A20cl), and posterior superior temporal sulcus (Table S1; Figure 1a). The four nodes that demonstrated the most substantial decrease in the SC were A38m (*P* = 0.0001, *R* = 4.000), A38l (*P* = 0.0001, *R* = 4.030), A21r (*P* = 0.00001, *R* = 4.560), and A20r (*P* = 0.00002, *R* = 4.490) (Table S1; Figure 1a–b). Among these nodes, A38m and A38l were situated in the STG, A21r was located in the middle temporal gyrus, and A20r was located in the inferior temporal gyrus. Specifically, after classification using the Yeo‐7 resting‐state network map, both A38m and A38l were found to belong to the frontoparietal network, whereas only node A21r was a part of the DMN. Notably, all these nodes reside within the anterior region of the temporal lobe.

Analysis of the relationship between seizure duration and SC revealed a significant negative linear correlation. Nodes exhibiting noteworthy correlations were predominantly distributed within the DMN and frontoparietal, visual, and subcortical networks (Table 2). Among these nodes, A38m (*p* = 0.004, *r* = 0.342), A38l (*p* = 0.002, *r* = 0.373), A21r (*p* = 0.010, *r* = 0.309), and A20r (*p* = 0.001, *r* = 0.391) demonstrated the strongest association with seizure duration and displayed the most substantial reduction in SC (Table 2; Figure 1b).

**TABLE 2:** All regional SC features that correlated with the seizure duration.

Label	Module	Region	Subregion	*p* value	*r* value^a^
94	**LNi**	**ITG**	**A20r**	**9.81E−04**	**−3.91E−01**
78	**LNi**	**STG**	**A38l**	**1.74E−03**	**−3.73E−01**
70	**LNi**	**STG**	**A38m**	**4.25E−03**	**−3.42E−01**
84	**DMNi**	**MTG**	**A21r**	**1.02E−02**	**−3.09E−01**
52	DMNi	OrG	A12/47l	2.04E−02	−2.81E−01
90	LNi	ITG	A20iv	2.09E−02	−2.80E−01
96	LNi	ITG	A20il	3.43E−02	−2.57E−01
136	VNi	IPL	A39c	4.92E−02	−2.39E−01
25	SMNc	MFG	A6vl	4.95E−02	2.39E−01
224	SCNi	BG	NAC	5.70E−02	−2.32E−01

Abbreviations: BG, basal ganglia; DMNi, default mode network ipsilateral; IPL, inferior parietal gyrus; ITG, inferior frontal gyrus; LNi, limbic network ipsilateral; MFG, middle frontal gyrus; MTG, middle temporal gyrus; OrG, orbital gyrus; SCNi, subcortical network ipsilateral; SMNc, somatosensory network; STG, superior temporal gyrus; VNi, visual network ipsilateral.

^a^ partial correlation analysis, non‐corrected.

### 3.3 Relationship Between Regional FC and Seizure Duration

**FIGURE 2:**
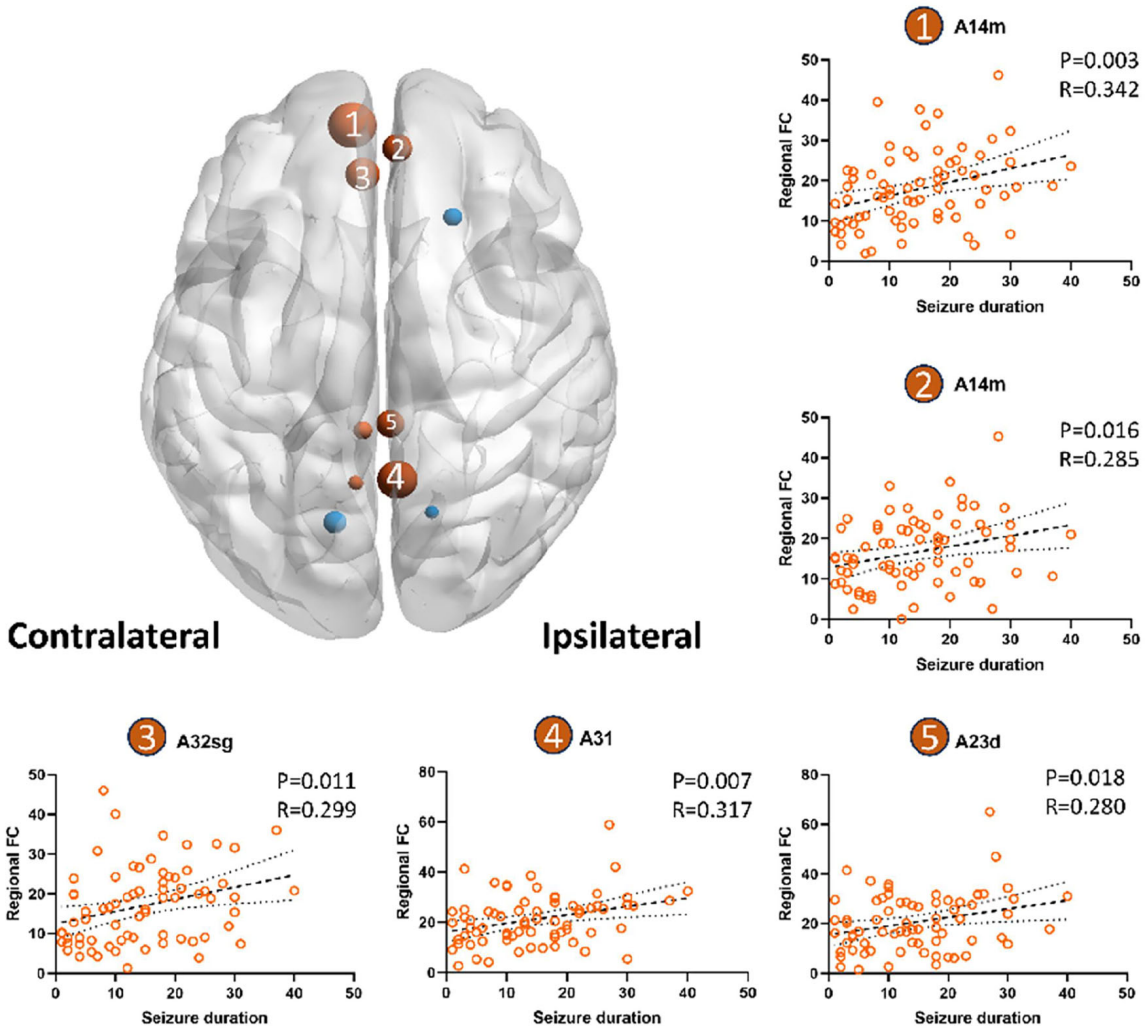
The five nodes exhibiting the strongest correlation between FC and duration of epileptic seizures are situated near the midline region (A14m, A14m, A31sg, A31, and A23d; these five nodes are all located within the default mode network), and the FC of these five nodes exhibits a positive correlation with epileptic seizure duration (partial correlation, *p* < 0.05, derived from linear regression analysis and presented for visualization purposes only).

Compared to the control group, the patient group exhibited significant reductions in FC within brain regions primarily associated with the frontoparietal, subcortical, default mode, and visual networks. Significantly reduced nodules were found in the hippocampus (cHipp, rHipp), parahippocampal gyrus (A35/36, TL, A28/34, A35/36c, TI), fusiform gyrus (A20rv), amygdala (lAmyg), inferior temporal gyrus (A20iv), middle temporal gyrus (A21r), insula (G, vId/vIg), basal ganglia (vmPu), and thalamus (Stha, PPtha, Otha, mPMtha) (Table S2; Figure S1). In our analysis of the relationship between seizure duration and FC, we observed a negative correlation with the seizure duration in most nodes. Classification using the Yeo‐7 resting‐state network map revealed that the nodes demonstrating significant correlations were predominantly distributed across the default mode, frontoparietal, subcortical, ventral attention, and visual networks (Table 3). However, in nodes near the midline, we observed a positive correlation with seizure duration. The five nodes that exhibited the strongest correlation with seizure duration were A14m (left hemisphere, *p* = 0.003; *r* = 0.342), A14m (right hemisphere, *p* = 0.016; *r* = 0.285), A32sg (*p* = 0.011; *r* = 0.299), A31 (*p* = 0.007; *r* = 0.317), and A23d (*p* = 0.018; *r* = 0.280) (Table 3; Figure 2). Specifically, A14m was located in the orbital gyrus, while A32sg and A23d were situated in the cingulate gyrus, along with A31, which resided in the anterior cingulate cortex region of interest within the five nodes belonging to the DMN.

**TABLE 3:** All regional FC features that correlated with the seizure duration.

Label	Module	Region	Subregion	*p* value	*r* value^a^
41	DMNc	OrG	A14m	3.49E−03	3.42E−01
154	DMNi	PCun	A31	7.11E−03	3.17E−01
187	DMNc	CG	A32sg	1.14E−02	2.99E−01
42	DMNi	OrG	A14m	1.62E−02	2.85E−01
176	DMNi	CG	A23d	1.79E−02	2.80E−01
94	LNi	ITG	A20r	1.87E−02	−2.78E−01
93	LNc	ITG	A20r	1.97E−02	−2.76E−01
97	DANc	ITG	A37vl	2.10E−02	−2.74E−01
45	LNc	OrG	A11l	2.30E−02	−2.70E−01
29	FPNc	IFG	A44d	2.51E−02	−2.66E−01
40	VANi	IFG	A44v	2.58E−02	−2.65E−01
151	VNc	PCun	dmPOS	2.64E−02	2.64E−01
4	FPNi	SFG	A8dl	3.53E−02	2.50E−01
175	DMNc	CG	A23d	3.56E−02	2.50E−01
212	SCNi	Amyg	mAmyg	3.92E−02	−2.45E−01
104	LNi	FuG	A20rv	3.95E−02	−2.45E−01
118	LNi	PhG	TI	4.04E−02	−2.44E−01
234	SCNi	Tha	mPMtha	4.06E−02	2.44E−01
63	DANc	PrG	A6cvl	4.10E−02	−2.43E−01
153	DMNc	PCun	A31	4.63E−02	2.37E−01
152	VNi	PCun	dmPOS	4.98E−02	2.34E−01

Abbreviations: Amyg, amygdala; CG, cingulate gyrus; DANc, dorsal attention network contralateral; DMNc, default mode network contralateral; DMNi, default mode network ipsilateral; FPNc, frontoparietal network contralateral; FPNi, frontoparietal network ipsilateral; FuG, fusiform gyrus; IFG, inferior frontal gyrus; ITG, inferior frontal gyrus; LNc, limbic network contralateral; LNi, limbic network ipsilateral; OrG, orbital gyrus; PCun, precuneus gyrus; PhG, parahippocampal gyrus, PrG, precentral gyrus; SCNi, subcortical network ipsilateral; SFG, superior frontal gyrus; Tha, thalamus; VANi, ventral attention network ipsilateral; VNc, visual network contralateral; VNi, visual network ipsilateral.

^a^ partial correlation analysis, non‐corrected.

## 4 Discussion

This study employed a connectome‐based approach to investigate the profiles of the structural and functional networks in individuals diagnosed with TLE. We aimed to explore the changes of functional and structural connectivity in patients with TLE and their relationship with seizure duration by automatically clustering and classifying connectivity profiles into predefined network modules. Our results revealed disruptions and decoupling in both SC and FC, as well as spatial heterogeneity between SC and FC. Nodes with a significant SC decline were concentrated in the limbic system close to the lesion, and these nodes were most significantly negatively correlated with the duration of epilepsy. The lymph nodes with significantly decreased FC were not only concentrated in the limbic system near the lesion but also in the thalamus and basal ganglia far from the lesion. In the whole brain, most of the FC nodes were negatively correlated with the duration of the seizure; however, in the midline region of the brain, far from the seizure focus, FC at certain nodes was positively correlated with seizure duration.

In studies investigating DTI in patients with TLE, researchers have identified reductions in local efficiency, FA, and connectivity compared to HC, primarily affecting ipsilateral temporal regions as well as bilateral frontal and parietal regions, the fornix, uncinate fasciculus, longitudinal fasciculi (superior and inferior), corticospinal tract, corpus callosum, and cingulate gyrus (Chiang et al. 2016; Concha et al. 2009; Nilsson et al. 2008; Otte et al. 2012). However, some studies have also reported compensatory reorganization of SC in TLE, manifested as increased clustering coefficients in the limbic system network and elevated nodal efficiency, degree centrality, and clustering coefficients in the insula, superior temporal regions, and thalamus (Bonilha et al. 2012). Similar phenomena were reported in another study, where researchers found that local connectivity in TLE patients increased by 85%–270% in the medial and lateral frontal cortex, insular cortex, posterior cingulate cortex, precuneus, and occipital cortex compared to healthy subjects (DeSalvo et al. 2014). These findings suggest complex reorganization mechanisms in the structural connectivity networks of TLE patients, which may also be related to relatively small sample sizes. Previous studies found that, compared with FC, the area of decreased nodes in SC was more limited, and the significantly decreased nodes were generally concentrated near the lesion (Galovic et al. 2019; Liu et al. 2016). In our study, we also observed a disruption of SC in patients with TLE, particularly evident in focal nodes located within the anterior temporal lobe. These nodes were predominantly situated proximal to the lesion site. We speculate that the SC alterations observed in our study may be due to the disruption of the white matter fiber microstructure due to prolonged interictal/ictal seizure activity, and the effects of seizures during brain development, excitotoxicity of neurotransmitters, and local ischemia may play an important role in this process (Fricker et al. 2018; Henshall 2007). Additionally, we identified a significant negative correlation between regional SC and longer seizure duration. This finding is consistent with previous research demonstrating an association between longer seizure duration, reduced SC integrity, and poorer prognosis in individuals with TLE (Horsley et al. 2022). The observed temporal decline in SC may reflect the dynamic process of white matter fiber structure degradation in the temporal lobe. The four nodes exhibiting the strongest correlation with seizure duration also exhibited the most significant SC decline. We hypothesized that this phenomenon might be associated with the vulnerability of certain nodes surrounding the lesion.

Simultaneously, and consistent with the SC findings, decreased connectivity was also observed in functional networks within ipsilateral temporal nodes—including the hippocampus, parahippocampal gyrus, fusiform gyrus, and amygdala. In addition, reduced FC was identified in ipsilateral thalamic and basal ganglia nodes, as well as in contralateral thalamic and temporal structures, potentially indicating possible pathways for seizure propagation. Similar findings have been reported in other studies. For instance, one study in pediatric TLE documented enhanced FC between the thalamus and mesiotemporal regions as well as the basal ganglia (Feng et al. 2025). Another stereoelectroencephalography (SEEG) study revealed strong connectivity between the thalamus and mesiotemporal regions during seizures in TLE (Soulier et al. 2023). These results suggest that subcortical structures, such as the thalamus and basal ganglia, may play an important role in the propagation of temporal lobe seizures. In our study, we found that nodes exhibiting significant decreases in FC were more widely dispersed than those in SC. The nodes demonstrating the most substantial decreases were distributed across the subcortical region, visual network, edge network, and DMN. FC represents the integration of information interactions among different brain regions, rather than direct fiber connections, exhibiting a wider range of decline. Consistent with previous studies, we observed that a more severe FC decline was associated with a longer seizure duration (Fleury et al. 2022; Haneef et al. 2015).

Interestingly, the FC of the five nodes close to the midline was positively correlated with seizure duration, all of which were located in the DMN, representing a large‐scale cortical system with ambiguous function. The core regions of the DMN include the ventral and dorsal medial prefrontal cortices, posterior cingulate cortex, and bilateral angular gyrus (Raichle 2015). The DMN is activated during spontaneous introspection and deactivated during goal‐directed behavior (De Luca et al. 2006; X. Wang et al. 2023). Furthermore, the DMN has been observed to interact with regions involved in cognitive control during complex working memory tasks (Gauffin et al. 2013; McCormick et al. 2013; Zhang et al. 2020). Studies have consistently reported that TLE affects both the structure and FC of the DMN, potentially explaining certain cognitive and psychiatric symptoms in patients with TLE (Hsiao et al. 2015; Zhou et al. 2019). A meta‐analysis also showed reduced FC in the posterior DMN (Jiang et al. 2022). These findings suggest that the DMN serves as a hub for integrating information across the cortex and may be more vulnerable to damage. Other studies found that the DMN exhibits plasticity and compensatory changes under pathological conditions. For instance, one study revealed an increase in structural–functional coupling within distant regions of the DMN, which intensified with the duration of TLE. This finding suggests compensatory mechanisms that counterbalance the decoupling caused by the loss of SC (Chiang et al. 2015). In another study, researchers found that when the FC between the unilateral DMN and the temporal lobe was impaired, the FC of the contralateral DMN was compensated (Haneef et al. 2015). We postulate that this increased FC in DMN nodes distant from the lesion may reflect a reconfiguration of compensatory processes. However, further research is warranted to investigate the potential underlying compensatory mechanisms driving hub reorganization, as this could indicate a pathological effect during disease progression.

However, our study has a number of limitations. First, the retrospective nature of this study may compromise the reliability of its findings. Additionally, the use of duration in years as a measure of seizure burden presents another limitation. Due to variations in seizure frequency among patients and over different time periods within patients, there might be inherent measurement error associated with this approach; however, the course of temporal lobe epilepsy is prolonged, and patients often cannot recall the frequency of seizures in the early stages. After medication, the seizure frequency is relatively low and not representative. We hope to further improve the collection of medical history related to seizure frequency in future studies. Moreover, the extensive history of antiepileptic drug use in patients with TLE could potentially act as a significant confounding factor when studying the natural progression of TLE brain networks. Finally, the relatively small size of our study cohort may restrict the generalizability and robustness of our findings; therefore, further validation through larger‐scale cohort studies is essential.

## 5 Conclusion

In patients with TLE, the brain exhibits inconsistent coupling patterns and heterogeneity in both functional and structural connectivity. Moreover, seizure duration exerts differential regulatory effects on structural and functional connectivity. These findings indicate that the evolutionary patterns of structural and functional connectivity may diverge in this patient population, potentially reflecting network reorganization.

## Author Contributions

**Yu Zhou**: Conceptualization; methodology; coding; data analysis; writing–original draft. **Liangchao Du**: Conceptualization; methodology; data analysis. **Fangfang Xie**: Methodology; coding; validation. **Dingyang Liu**: Validation; conceptualization. **Zhuanyi Yang**: Conceptualization; validation; data curation. **Chao Deng**: Data collection. **Chunyao Zhou**: Conceptualization; coding; methodology; writing–review & editing. **Zhiquan Yang**: Supervision; conceptualization; validation; methodology; writing–review & editing; writing–original draft; funding acquisition.

## Ethics Statement

The study has been reviewed and approved by the Ethics Committee of Xiangya Hospital, Central South University.

## Consent

Written informed consent was obtained from all participants prior to their involvement in this study.

## Conflicts of Interest

The authors declare no conflict of interest.

## Peer Review

The peer review history for this article is available at https://publons.com/publon/10.1002/brb3.70935

## Supporting information




**Supplementary Table 1**: brb370935‐sup‐0001‐SuppMat.docx

## Data Availability

The data and codes utilized or analyzed in this study are accessible from the corresponding author upon request.

## References

[brb370935-bib-0001] Bonilha, L. , T. Nesland , G. U. Martz , et al. 2012. “Medial Temporal Lobe Epilepsy Is Associated With Neuronal Fibre Loss and Paradoxical Increase in Structural Connectivity of Limbic Structures.” Journal of Neurology, Neurosurgery, and Psychiatry 83, no. 9: 903–909. 10.1136/jnnp-2012-302476. 22764263 PMC3415309

[brb370935-bib-0002] Chiang, S. , H. S. Levin , E. Wilde , and Z. Haneef . 2016. “White Matter Structural Connectivity Changes Correlate With Epilepsy Duration in Temporal Lobe Epilepsy.” Epilepsy Research 120: 37–46. 10.1016/j.eplepsyres.2015.12.002. 26709881 PMC4740226

[brb370935-bib-0003] Chiang, S. , J. M. Stern , J. Engel Jr. , and Z. Haneef . 2015. “Structural–Functional Coupling Changes in Temporal Lobe Epilepsy.” Brain Research 1616: 45–57. 10.1016/j.brainres.2015.04.052. 25960346 PMC4892844

[brb370935-bib-0004] Concha, L. , C. Beaulieu , D. L. Collins , and D. W. Gross . 2009. “White‐Matter Diffusion Abnormalities in Temporal‐Lobe Epilepsy With and Without Mesial Temporal Sclerosis.” Journal of Neurology, Neurosurgery, and Psychiatry 80, no. 3: 312–319. 10.1136/jnnp.2007.139287. 18977826

[brb370935-bib-0005] De Luca, M. , C. F. Beckmann , N. De Stefano , P. M. Matthews , and S. M. Smith . 2006. “fMRI Resting State Networks Define Distinct Modes of Long‐Distance Interactions in the Human Brain.” NeuroImage 29, no. 4: 1359–1367. 10.1016/j.neuroimage.2005.08.035. 16260155

[brb370935-bib-0006] DeSalvo, M. N. , L. Douw , N. Tanaka , C. Reinsberger , and S. M. Stufflebeam . 2014. “Altered Structural Connectome in Temporal Lobe Epilepsy.” Radiology 270, no. 3: 842–848. 10.1148/radiol.13131044. 24475828 PMC4263659

[brb370935-bib-0007] Feng, X. , H. Xie , R. J. Piper , et al. 2025. “Altered Thalamic Connectivity Patterns in Pediatric Temporal Lobe Epilepsy: A Gradient Mapping Study.” Epilepsia. 10.1111/epi.18515. 40608009

[brb370935-bib-0008] Fleury, M. , S. Buck , L. P. Binding , et al. 2022. “Episodic Memory Network Connectivity in Temporal Lobe Epilepsy.” Epilepsia 63, no. 10: 2597–2622. 10.1111/epi.17370. 35848050 PMC9804196

[brb370935-bib-0009] Foesleitner, O. , B. Sigl , V. Schmidbauer , et al. 2021. “Language Network Reorganization Before and After Temporal Lobe Epilepsy Surgery.” Journal of Neurosurgery 134, no. 6: 1694–1702. 10.3171/2020.4.Jns193401. 32619977

[brb370935-bib-0010] Fricker, M. , A. M. Tolkovsky , V. Borutaite , M. Coleman , and G. C. Brown . 2018. “Neuronal Cell Death.” Physiological Reviews 98, no. 2: 813–880. 10.1152/physrev.00011.2017. 29488822 PMC5966715

[brb370935-bib-0011] Galovic, M. , V. Q. H. van Dooren , T. S. Postma , et al. 2019. “Progressive Cortical Thinning in Patients with Focal Epilepsy.” JAMA Neurology 76, no. 10: 1230–1239. 10.1001/jamaneurol.2019.1708. 31260004 PMC6604082

[brb370935-bib-0012] Gauffin, H. , H. van Ettinger‐Veenstra , A. M. Landtblom , et al. 2013. “Impaired Language Function in Generalized Epilepsy: Inadequate Suppression of the Default Mode Network.” Epilepsy & Behavior 28, no. 1: 26–35. 10.1016/j.yebeh.2013.04.001. 23648277

[brb370935-bib-0013] Haneef, Z. , S. Chiang , H. J. Yeh , J. Engel Jr. , and J. M. Stern . 2015. “Functional Connectivity Homogeneity Correlates With Duration of Temporal Lobe Epilepsy.” Epilepsy & Behavior 46: 227–233. 10.1016/j.yebeh.2015.01.025. 25873437 PMC4458387

[brb370935-bib-0014] Henshall, D. C. 2007. “Apoptosis Signalling Pathways in Seizure‐induced Neuronal Death and Epilepsy.” Biochemical Society Transactions 35: 421–423. 10.1042/bst0350421. 17371290

[brb370935-bib-0015] Horsley, J. J. , G. M. Schroeder , R. H. Thomas , et al. 2022. “Volumetric and Structural Connectivity Abnormalities Co‐Localise in TLE.” NeuroImage: Clinical 35: 103105. 10.1016/j.nicl.2022.103105. 35863179 PMC9421455

[brb370935-bib-0016] Hsiao, F. J. , H. Y. Yu , W. T. Chen , et al. 2015. “Increased Intrinsic Connectivity of the Default Mode Network in Temporal Lobe Epilepsy: Evidence From Resting‐State MEG Recordings.” PLoS ONE 10, no. 6: e0128787. 10.1371/journal.pone.0128787. 26035750 PMC4452781

[brb370935-bib-0017] Jiang, S. , H. Li , L. Liu , D. Yao , and C. Luo . 2022. “Voxel‐Wise Functional Connectivity of the Default Mode Network in Epilepsies: A Systematic Review and Meta‐Analysis.” Current Neuropharmacology 20, no. 1: 254–266. 10.2174/1570159x19666210325130624. 33823767 PMC9199542

[brb370935-bib-0018] Kelly, C. , B. B. Biswal , R. C. Craddock , F. X. Castellanos , and M. P. Milham . 2012. “Characterizing Variation in the Functional Connectome: Promise and Pitfalls.” Trends in Cognitive Sciences 16, no. 3: 181–188. 10.1016/j.tics.2012.02.001. 22341211 PMC3882689

[brb370935-bib-0019] Li, X. , Y. Jiang , W. Li , et al. 2022. “Disrupted Functional Connectivity in White Matter Resting‐State Networks in Unilateral Temporal Lobe Epilepsy.” Brain Imaging and Behavior 16, no. 1: 324–335. 10.1007/s11682-021-00506-8. 34478055

[brb370935-bib-0020] Liang, X. , X. Pang , J. Zhao , et al. 2021. “Altered Static and Dynamic Functional Network Connectivity in Temporal Lobe Epilepsy With Different Disease Duration and Their Relationships With Attention.” Journal of Neuroscience Research 99, no. 10: 2688–2705. 10.1002/jnr.24915. 34269468

[brb370935-bib-0021] Liu, M. , B. C. Bernhardt , A. Bernasconi , and N. Bernasconi . 2016. “Gray Matter Structural Compromise Is Equally Distributed in Left and Right Temporal Lobe Epilepsy.” Human Brain Mapping 37, no. 2: 515–524. 10.1002/hbm.23046. 26526187 PMC6867333

[brb370935-bib-0022] McCormick, C. , M. Quraan , M. Cohn , T. A. Valiante , and M. P. McAndrews . 2013. “Default Mode Network Connectivity Indicates Episodic Memory Capacity in Mesial Temporal Lobe Epilepsy.” Epilepsia 54, no. 5: 809–818. 10.1111/epi.12098. 23360362

[brb370935-bib-0023] Morgan, V. L. , B. Abou‐Khalil , and B. P. Rogers . 2015. “Evolution of Functional Connectivity of Brain Networks and Their Dynamic Interaction in Temporal Lobe Epilepsy.” Brain Connectivity 5, no. 1: 35–44. 10.1089/brain.2014.0251. 24901036 PMC4313394

[brb370935-bib-0024] Morgan, V. L. , C. Chang , D. J. Englot , and B. P. Rogers . 2020. “Temporal Lobe Epilepsy Alters Spatio‐Temporal Dynamics of the Hippocampal Functional Network.” NeuroImage: Clinical 26: 102254. 10.1016/j.nicl.2020.102254. 32251905 PMC7132094

[brb370935-bib-0025] Nilsson, D. , C. Go , J. T. Rutka , et al. 2008. “Bilateral Diffusion Tensor Abnormalities of Temporal Lobe and Cingulate Gyrus White Matter in Children With Temporal Lobe Epilepsy.” Epilepsy Research 81, no. 2–3: 128–135. 10.1016/j.eplepsyres.2008.05.002. 18595664

[brb370935-bib-0026] Otte, W. M. , P. van Eijsden , J. W. Sander , J. S. Duncan , R. M. Dijkhuizen , and K. P. Braun . 2012. “A Meta‐Analysis of White Matter Changes in Temporal Lobe Epilepsy as Studied With Diffusion Tensor Imaging.” Epilepsia 53, no. 4: 659–667. 10.1111/j.1528-1167.2012.03426.x. 22379949

[brb370935-bib-0027] Raichle, M. E. 2015. “The Brain's Default Mode Network.” Annual Review of Neuroscience 38: 433–447. 10.1146/annurev-neuro-071013-014030. 25938726

[brb370935-bib-0028] Smith, S. M. , D. Vidaurre , C. F. Beckmann , et al. 2013. “Functional Connectomics From Resting‐State fMRI.” Trends in Cognitive Sciences 17, no. 12: 666–682. 10.1016/j.tics.2013.09.016. 24238796 PMC4004765

[brb370935-bib-0029] Soulier, H. , F. Pizzo , A. Jegou , et al. 2023. “The Anterior and Pulvinar Thalamic Nuclei Interactions in Mesial Temporal Lobe Seizure Networks.” Clinical Neurophysiology 150: 176–183. 10.1016/j.clinph.2023.03.016. 37075682

[brb370935-bib-0030] Trimmel, K. , S. B. Vos , L. Caciagli , et al. 2021. “Decoupling of Functional and Structural Language Networks in Temporal Lobe Epilepsy.” Epilepsia 62, no. 12: 2941–2954. 10.1111/epi.17098. 34642939 PMC8776336

[brb370935-bib-0031] Voets, N. L. , C. F. Beckmann , D. M. Cole , S. Hong , A. Bernasconi , and N. Bernasconi . 2012. “Structural Substrates for Resting Network Disruption in Temporal Lobe Epilepsy.” Brain 135, no. Pt 8: 2350–2357. 10.1093/brain/aws137. 22669081

[brb370935-bib-0032] Wan, X. , P. Zhang , W. Wang , et al. 2023. “Abnormal Brain Functional Network Dynamics in Sleep‐Related Hypermotor Epilepsy.” CNS Neuroscience & Therapeutics 29, no. 2: 659–668. 10.1111/cns.14048. 36510701 PMC9873504

[brb370935-bib-0033] Wang, J. , X. Wang , M. Xia , X. Liao , A. Evans , and Y. He . 2015. “GRETNA: A Graph Theoretical Network Analysis Toolbox for Imaging Connectomics.” Frontiers in Human Neuroscience 9: 386. 10.3389/fnhum.2015.00386. 26175682 PMC4485071

[brb370935-bib-0034] Wang, X. , D. Lin , C. Zhao , et al. 2023. “Abnormal Metabolic Connectivity in Default Mode Network of Right Temporal Lobe Epilepsy.” Frontiers in Neuroscience 17: 1011283. 10.3389/fnins.2023.1011283. 37034164 PMC10076532

[brb370935-bib-0035] Yeo, B. T. , F. M. Krienen , J. Sepulcre , et al. 2011. “The Organization of the Human Cerebral Cortex Estimated by Intrinsic Functional Connectivity.” Journal of Neurophysiology 106, no. 3: 1125–1165. 10.1152/jn.00338.2011. 21653723 PMC3174820

[brb370935-bib-0036] Zhang, Z. , X. Zhou , J. Liu , L. Qin , W. Ye , and J. Zheng . 2020. “Aberrant Executive Control Networks and Default Mode Network in Patients With Right‐Sided Temporal Lobe Epilepsy: A Functional and Effective Connectivity Study.” International Journal of Neuroscience 130, no. 7: 683–693. 10.1080/00207454.2019.1702545. 31851554

[brb370935-bib-0037] Zhou, X. , Z. Zhang , J. Liu , L. Qin , and J. Zheng . 2019. “Aberrant Topological Organization of the Default Mode Network in Temporal Lobe Epilepsy Revealed by Graph‐Theoretical Analysis.” Neuroscience Letters 708: 134351. 10.1016/j.neulet.2019.134351. 31247225

