# Supplementary material for: Disparities in Connectivity Profiles Between Structural and Functional Networks in Patients With Temporal Lobe Epilepsy
Source: Brain Behav. 2025 Sep 27;15(10):e70935. doi: 10.1002/brb3.70935 (PMC12475998; doi:10.1002/brb3.70935)
Supplement: Supplementary file 1 — Supplementary Table 1: brb370935‐sup‐0001‐SuppMat.docx [file BRB3-15-e70935-s001.docx]

**Supplementary Table 1: Significant difference between HC and TLE on regional SC**

| node | module | region | subregion | P value* | T value |
| --- | --- | --- | --- | --- | --- |
| 84 | **DMNi** | **MTG** | **A21r** | **1.29E-05** | **4.56E+00** |
| 94 | **LNi** | **ITG** | **A20r** | **1.70E-05** | **4.49E+00** |
| 78 | **LNi** | **STG** | **A38l** | **1.01E-04** | **4.03E+00** |
| 70 | **LNi** | **STG** | **A38m** | **1.13E-04** | **4.00E+00** |
| 96 | LNi | ITG | A20il | 2.91E-04 | 3.74E+00 |
| 122 | DMNi | pSTS | rpSTS | 3.22E-04 | 3.71E+00 |
| 82 | FPNi | MTG | A21c | 3.25E-04 | 3.71E+00 |
| 100 | FPNi | ITG | A20cl | 1.08E-03 | 3.35E+00 |

*FDR corrected; DMNi, default mode network ipsilateral; LNi, limbic network ipsilateral; FPNi, frontoparietal network ipsilateral; MTG, middle temporal gyrus; ITG, inferior frontal gyrus; STG, superior temporal gyrus; pSTS, posterior superior temporal sulcus

**Supplementary Table 2: Significant difference between HC and TLE on regional FC**

| node | module |  |  | region | | | subregion | | P value* | | T value |
| --- | --- | --- | --- | --- | --- | --- | --- | --- | --- | --- | --- |
| 218 | SCNi |  |  | Hipp | | cHipp | | | 1.95E-08 | | 6.04 |
| 110 | LNi |  |  | PhG | A35/36r | | | 6.76E-08 | | 5.78 | |
| 114 | VNi |  |  | PhG | TL | | | 1.46E-07 | | 5.61 | |
| 104 | LNi |  |  | FuG | A20rv | | | 3.36E-07 | | 5.42 | |
| 116 | LNi |  |  | PhG | A28/34 | | | 2.33E-06 | | 4.98 | |
| 113 | VNc |  |  | PhG | TL | | | 1.17E-05 | | 4.58 | |
| 103 | LNc |  |  | FuG | A20rv | | | 2.48E-05 | | 4.40 | |
| 214 | SCNi |  |  | Amyg | lAmyg | | | 7.53E-05 | | 4.11 | |
| 216 | SCNi |  |  | Hipp | rHipp | | | 1.18E-04 | | 3.99 | |
| 90 | LNi |  |  | ITG | A20iv | | | 1.48E-04 | | 3.93 | |
| 115 | LNc |  |  | PhG | A28/34 | | | 2.78E-04 | | 3.75 | |
| 84 | DMNc |  |  | MTG | A21r | | | 2.93E-04 | | 3.74 | |
| 89 | LNc |  |  | ITG | A20iv | | | 3.87E-04 | | 3.66 | |
| 111 | LNc |  |  | PhG | A35/36c | | | 4.45E-04 | | 3.62 | |
| 170 | VNc |  |  | INS | vId/vIg | | | 4.48E-04 | | 3.62 | |
| 215 | SCNc |  |  | Hipp | rHipp | | | 5.60E-04 | | 3.55 | |
| 112 | VNi |  |  | PhG | A35/36c | | | 9.03E-04 | | 3.41 | |
| 118 | LNi |  |  | PhG | TI | | | 1.16E-03 | | 3.33 | |
| 236 | SCNi |  |  | Tha | Stha | | | 1.44E-03 | | 3.27 | |
| 117 | LNc |  |  | PhG | TI | | | 1.82E-03 | | 3.19 | |
| 226 | SCNi |  |  | BG | vmPu | | | 2.06E-03 | | 3.15 | |
| 235 | SCNc |  |  | Tha | Stha | | | 2.07E-03 | | 3.15 | |
| 164 | VNc |  |  | INS | G | | | 2.22E-03 | | 3.13 | |
| 239 | SCNc |  |  | Tha | PPtha | | | 3.19E-03 | | 3.01 | |
| 242 | SCNi |  |  | Tha | Otha | | | 3.64E-03 | | 2.97 | |
| 233 | SCNc |  |  | Tha | mPMtha | | | 5.12E-03 | | 2.85 | |

*FDR corrected; SCNi, subcortical network ipsilateral; LNi, limbic network ipsilateral; VNi ,visual network ipsilateral; VNc, visual network contralateral; LNc, limbic network contralateral; DMNc, default mode network contralateral; SCNc, subcortical network contralateral;Hipp, hippocampu; PhG, parahippocampal gyrus; FuG, fusiform gyrus; Amyg, amygdala; ITG, inferior frontal gyrus; MTG, middle temporal gyrus; INS, insula; Tha, thalamu; BG, basal ganglia

**Supplementary Table 3: All nodes and their belonging networks.**

| Label | subregion name | region |  | Network assignment | |
| --- | --- | --- | --- | --- | --- |
| 1 | A8m | SFG_L_7_1 |  | Frontoparietal_L | |
| 2 | A8m | SFG_R_7_1 |  | Ventral Attention_R | |
| 3 | A8dl | SFG_L_7_2 |  | Default_L | |
| 4 | A8dl | SFG_R_7_2 |  | Frontoparietal_R | |
| 5 | A9l | SFG_L_7_3 |  | Default_L | |
| 6 | A9l | SFG_R_7_3 |  | Default_R | |
| 7 | A6dl | SFG_L_7_4 |  | Dorsal Attention_L | |
| 8 | A6dl | SFG_R_7_4 |  | Dorsal Attention_R | |
| 9 | A6m | SFG_L_7_5 |  | Somatomotor_L | |
| 10 | A6m | SFG_R_7_5 |  | Somatomotor_R | |
| 11 | A9m | SFG_L_7_6 |  | Default_L | |
| 12 | A9m | SFG_R_7_6 |  | Frontoparietal_R | |
| 13 | A10m | SFG_L_7_7 |  | Default_L | |
| 14 | A10m | SFG_R_7_7 |  | Default_R | |
| 15 | A9/46d | MFG_L_7_1 |  | Ventral Attention_L | |
| 16 | A9/46d | MFG_R_7_1 |  | Frontoparietal_R | |
| 17 | IFJ | MFG_L_7_2 |  | Frontoparietal_L | |
| 18 | IFJ | MFG_R_7_2 |  | Frontoparietal_R | |
| 19 | A46 | MFG_L_7_3 |  | Frontoparietal_L | |
| 20 | A46 | MFG_R_7_3 |  | Frontoparietal_R | |
| 21 | A9/46v | MFG_L_7_4 |  | Frontoparietal_L | |
| 22 | A9/46v | MFG_R_7_4 |  | Frontoparietal_R | |
| 23 | A8vl | MFG_L_7_5 |  | Default_L | |
| 24 | A8vl | MFG_R_7_5 |  | Frontoparietal_R | |
| 25 | A6vl | MFG_L_7_6 |  | Dorsal Attention_L | |
| 26 | A6vl | MFG_R_7_6 |  | Dorsal Attention_R | |
| 27 | A10l | MFG_L_7_7 |  | Limbic_L | |
| 28 | A10l | MFG_R_7_7 |  | Frontoparietal_R | |
| 29 | A44d | IFG_L_6_1 |  | Frontoparietal_L | |
| 30 | A44d | IFG_R_6_1 |  | Dorsal Attention_R | |
| 31 | IFS | IFG_L_6_2 |  | Frontoparietal_L | |
| 32 | IFS | IFG_R_6_2 |  | Frontoparietal_R | |
| 33 | A45c | IFG_L_6_3 |  | Default_L | |
| 34 | A45c | IFG_R_6_3 |  | Default_R | |
| 35 | A45r | IFG_L_6_4 |  | Default_L | |
| 36 | A45r | IFG_R_6_4 |  | Frontoparietal_R | |
| 37 | A44op | IFG_L_6_5 |  | Ventral Attention_L | |
| 38 | A44op | IFG_R_6_5 |  | | Ventral Attention_R |
| 39 | A44v | IFG_L_6_6 |  | | Ventral Attention_L |
| 40 | A44v | IFG_R_6_6 |  | | Ventral Attention_R |

**Supplementary Table 3: All nodes and their belonging networks. (continued)**

| Label | subregion name | region |  | Network assignment |
| --- | --- | --- | --- | --- |
| 41 | A14m | OrG_L_6_1 |  | Default_L |
| 42 | A14m | OrG_R_6_1 |  | Default_R |
| 43 | A12/47o | OrG_L_6_2 |  | Default_L |
| 44 | A12/47o | OrG_R_6_2 |  | Default_R |
| 45 | A11l | OrG_L_6_3 |  | Limbic_L |
| 46 | A11l | OrG_R_6_3 |  | Frontoparietal_R |
| 47 | A11m | OrG_L_6_4 |  | Limbic_L |
| 48 | A11m | OrG_R_6_4 |  | Limbic_R |
| 49 | A13 | OrG_L_6_5 |  | Limbic_L |
| 50 | A13 | OrG_R_6_5 |  | Limbic_R |
| 51 | A12/47l | OrG_L_6_6 |  | Default_L |
| 52 | A12/47l | OrG_R_6_6 |  | Default_R |
| 53 | A4hf | PrG_L_6_1 |  | Somatomotor_L |
| 54 | A4hf | PrG_R_6_1 |  | Somatomotor_R |
| 55 | A6cdl | PrG_L_6_2 |  | Dorsal Attention_L |
| 56 | A6cdl | PrG_R_6_2 |  | Dorsal Attention_R |
| 57 | A4ul | PrG_L_6_3 |  | Somatomotor_L |
| 58 | A4ul | PrG_R_6_3 |  | Somatomotor_R |
| 59 | A4t | PrG_L_6_4 |  | Somatomotor_L |
| 60 | A4t | PrG_R_6_4 |  | Somatomotor_R |
| 61 | A4tl | PrG_L_6_5 |  | Ventral Attention_L |
| 62 | A4tl | PrG_R_6_5 |  | Ventral Attention_R |
| 63 | A6cvl | PrG_L_6_6 |  | Dorsal Attention_L |
| 64 | A6cvl | PrG_R_6_6 |  | Dorsal Attention_R |
| 65 | A1/2/3ll | PCL_L_2_1 |  | Ventral Attention_L |
| 66 | A1/2/3ll | PCL_R_2_1 |  | Somatomotor_R |
| 67 | A4ll | PCL_L_2_2 |  | Somatomotor_L |
| 68 | A4ll | PCL_R_2_2 |  | Somatomotor_R |
| 69 | A38m | STG_L_6_1 |  | Limbic_L |
| 70 | A38m | STG_R_6_1 |  | Limbic_R |
| 71 | A41/42 | STG_L_6_2 |  | Somatomotor_L |
| 72 | A41/42 | STG_R_6_2 |  | Somatomotor_R |
| 73 | TE1.0/TE1.2 | STG_L_6_3 |  | Somatomotor_L |
| 74 | TE1.0/TE1.2 | STG_R_6_3 |  | Somatomotor_R |
| 75 | A22c | STG_L_6_4 |  | Somatomotor_L |
| 76 | A22c | STG_R_6_4 |  | Somatomotor_R |
| 77 | A38l | STG_L_6_5 |  | Limbic_L |
| 78 | A38l | STG_R_6_5 |  | Limbic_R |
| 79 | A22r | STG_L_6_6 |  | Default_L |
| 80 | A22r | STG_R_6_6 |  | Default_R |

**Supplementary Table 3: All nodes and their belonging networks. (continued)**

| Label | subregion name | region |  | Network assignment |
| --- | --- | --- | --- | --- |
| 81 | A21c | MTG_L_4_1 |  | Default_L |
| 82 | A21c | MTG_R_4_1 |  | Frontoparietal_R |
| 83 | A21r | MTG_L_4_2 |  | Default_L |
| 84 | A21r | MTG_R_4_2 |  | Default_R |
| 85 | A37dl | MTG_L_4_3 |  | Dorsal Attention_L |
| 86 | A37dl | MTG_R_4_3 |  | Dorsal Attention_R |
| 87 | aSTS | MTG_L_4_4 |  | Default_L |
| 88 | aSTS | MTG_R_4_4 |  | Default_R |
| 89 | A20iv | ITG_L_7_1 |  | Limbic_L |
| 90 | A20iv | ITG_R_7_1 |  | Limbic_R |
| 91 | A37elv | ITG_L_7_2 |  | Dorsal Attention_L |
| 92 | A37elv | ITG_R_7_2 |  | Dorsal Attention_R |
| 93 | A20r | ITG_L_7_3 |  | Limbic_L |
| 94 | A20r | ITG_R_7_3 |  | Limbic_R |
| 95 | A20il | ITG_L_7_4 |  | Default_L |
| 96 | A20il | ITG_R_7_4 |  | Limbic_R |
| 97 | A37vl | ITG_L_7_5 |  | Dorsal Attention_L |
| 98 | A37vl | ITG_R_7_5 |  | Dorsal Attention_R |
| 99 | A20cl | ITG_L_7_6 |  | Frontoparietal_L |
| 100 | A20cl | ITG_R_7_6 |  | Frontoparietal_R |
| 101 | A20cv | ITG_L_7_7 |  | Limbic_L |
| 102 | A20cv | ITG_R_7_7 |  | Limbic_R |
| 103 | A20rv | FuG_L_3_1 |  | Limbic_L |
| 104 | A20rv | FuG_R_3_1 |  | Limbic_R |
| 105 | A37mv | FuG_L_3_2 |  | Visual_L |
| 106 | A37mv | FuG_R_3_2 |  | Visual_R |
| 107 | A37lv | FuG_L_3_3 |  | Dorsal Attention_L |
| 108 | A37lv | FuG_R_3_3 |  | Visual_R |
| 109 | A35/36r | PhG_L_6_1 |  | Limbic_L |
| 110 | A35/36r | PhG_R_6_1 |  | Limbic_R |
| 111 | A35/36c | PhG_L_6_2 |  | Limbic_L |
| 112 | A35/36c | PhG_R_6_2 |  | Visual_R |
| 113 | TL | PhG_L_6_3 |  | Visual_L |
| 114 | TL | PhG_R_6_3 |  | Visual_R |
| 115 | A28/34 | PhG_L_6_4 |  | Limbic_L |
| 116 | A28/34 | PhG_R_6_4 |  | Limbic_R |
| 117 | TI | PhG_L_6_5 |  | Limbic_L |
| 118 | TI | PhG_R_6_5 |  | Limbic_R |
| 119 | TH | PhG_L_6_6 |  | Visual_L |
| 120 | TH | PhG_R_6_6 |  | Visual_R |

**Supplementary Table 3: All nodes and their belonging networks. (continued)**

| Label | subregion name | region |  | Network assignment |
| --- | --- | --- | --- | --- |
| 121 | rpSTS | pSTS_L_2_1 |  | Default_L |
| 122 | rpSTS | pSTS_R_2_1 |  | Default_R |
| 123 | cpSTS | pSTS_L_2_2 |  | Ventral Attention_L |
| 124 | cpSTS | pSTS_R_2_2 |  | Ventral Attention_R |
| 125 | A7r | SPL_L_5_1 |  | Dorsal Attention_L |
| 126 | A7r | SPL_R_5_1 |  | Dorsal Attention_R |
| 127 | A7c | SPL_L_5_2 |  | Dorsal Attention_L |
| 128 | A7c | SPL_R_5_2 |  | Dorsal Attention_R |
| 129 | A5l | SPL_L_5_3 |  | Dorsal Attention_L |
| 130 | A5l | SPL_R_5_3 |  | Dorsal Attention_R |
| 131 | A7pc | SPL_L_5_4 |  | Somatomotor_L |
| 132 | A7pc | SPL_R_5_4 |  | Somatomotor_R |
| 133 | A7ip | SPL_L_5_5 |  | Dorsal Attention_L |
| 134 | A7ip | SPL_R_5_5 |  | Dorsal Attention_R |
| 135 | A39c | IPL_L_6_1 |  | Visual_L |
| 136 | A39c | IPL_R_6_1 |  | Visual_R |
| 137 | A39rd | IPL_L_6_2 |  | Frontoparietal_L |
| 138 | A39rd | IPL_R_6_2 |  | Frontoparietal_R |
| 139 | A40rd | IPL_L_6_3 |  | Dorsal Attention_L |
| 140 | A40rd | IPL_R_6_3 |  | Dorsal Attention_R |
| 141 | A40c | IPL_L_6_4 |  | Default_L |
| 142 | A40c | IPL_R_6_4 |  | Frontoparietal_R |
| 143 | A39rv | IPL_L_6_5 |  | Dorsal Attention_L |
| 144 | A39rv | IPL_R_6_5 |  | Default_R |
| 145 | A40rv | IPL_L_6_6 |  | Somatomotor_L |
| 146 | A40rv | IPL_R_6_6 |  | Somatomotor_R |
| 147 | A7m | PCun_L_4_1 |  | Frontoparietal_L |
| 148 | A7m | PCun_R_4_1 |  | Frontoparietal_R |
| 149 | A5m | PCun_L_4_2 |  | Somatomotor_L |
| 150 | A5m | PCun_R_4_2 |  | Dorsal Attention_R |
| 151 | dmPOS | PCun_L_4_3 |  | Visual_L |
| 152 | dmPOS | PCun_R_4_3 |  | Visual_R |
| 153 | A31 | PCun_L_4_4 |  | Default_L |
| 154 | A31 | PCun_R_4_4 |  | Default_R |
| 155 | A1/2/3ulhf | PoG_L_4_1 |  | Somatomotor_L |
| 156 | A1/2/3ulhf | PoG_R_4_1 |  | Somatomotor_R |
| 157 | A1/2/3tonIa | PoG_L_4_2 |  | Somatomotor_L |
| 158 | A1/2/3tonIa | PoG_R_4_2 |  | Somatomotor_R |
| 159 | A2 | PoG_L_4_3 |  | Dorsal Attention_L |
| 160 | A2 | PoG_R_4_3 |  | Somatomotor_R |

**Supplementary Table 3: All nodes and their belonging networks. (continued)**

| Label | Subregion name | region |  | | | Network assignment |
| --- | --- | --- | --- | --- | --- | --- |
| 161 | A1/2/3tru | PoG_L_4_4 | |  | Somatomotor_L | |
| 162 | A1/2/3tru | PoG_R_4_4 | |  | Somatomotor_R | |
| 163 | G | INS_L_6_1 | |  | Somatomotor_L | |
| 164 | G | INS_R_6_1 | |  | Somatomotor_R | |
| 165 | vIa | INS_L_6_2 | |  | subcortical_L | |
| 166 | vIa | INS_R_6_2 | |  | Frontoparietal_R | |
| 167 | dIa | INS_L_6_3 | |  | Ventral Attention_L | |
| 168 | dIa | INS_R_6_3 | |  | Ventral Attention_R | |
| 169 | vId/vIg | INS_L_6_4 | |  | Ventral Attention_L | |
| 170 | vId/vIg | INS_R_6_4 | |  | Ventral Attention_R | |
| 171 | dIg | INS_L_6_5 | |  | Somatomotor_L | |
| 172 | dIg | INS_R_6_5 | |  | Somatomotor_R | |
| 173 | dId | INS_L_6_6 | |  | Ventral Attention_L | |
| 174 | dId | INS_R_6_6 | |  | Ventral Attention_R | |
| 175 | A23d | CG_L_7_1 | |  | Default_L | |
| 176 | A23d | CG_R_7_1 | |  | Default_R | |
| 177 | A24rv | CG_L_7_2 | |  | subcortical_L | |
| 178 | A24rv | CG_R_7_2 | |  | subcortical_R | |
| 179 | A32p | CG_L_7_3 | |  | Default_L | |
| 180 | A32p | CG_R_7_3 | |  | Ventral Attention_R | |
| 181 | A23v | CG_L_7_4 | |  | Default_L | |
| 182 | A23v | CG_R_7_4 | |  | Visual_R | |
| 183 | A24cd | CG_L_7_5 | |  | Ventral Attention_L | |
| 184 | A24cd | CG_R_7_5 | |  | Ventral Attention_R | |
| 185 | A23c | CG_L_7_6 | |  | Ventral Attention_L | |
| 186 | A23c | CG_R_7_6 | |  | Ventral Attention_R | |
| 187 | A32sg | CG_L_7_7 | |  | Default_L | |
| 188 | A32sg | CG_R_7_7 | |  | Default_R | |
| 189 | cLinG | MVOcC_L_5_1 | |  | VisuaL_L | |
| 190 | cLinG | MVOcC_R_5_1 | |  | Visual_R | |
| 191 | rCunG | MVOcC_L_5_2 | |  | VisuaL_L | |
| 192 | rCunG | MVOcC_R_5_2 | |  | Visual_R | |
| 193 | cCunG | MVOcC_L_5_3 | |  | VisuaL_L | |
| 194 | cCunG | MVOcC_R_5_3 | |  | Visual_R | |
| 195 | rLinG | MVOcC_L_5_4 | |  | VisuaL_L | |
| 196 | rLinG | MVOcC_R_5_4 | |  | Visual_R | |
| 197 | vmPOS | MVOcC_L_5_5 | |  | VisuaL_L | |
| 198 | vmPOS | MVOcC_R_5_5 | |  | Visual_R | |
| 199 | mOccG | LOcC_L_4_1 | |  | VisuaL_L | |
| 200 | mOccG | LOcC_R_4_1 | |  | Visual_R | |

**Supplementary Table 3: All nodes and their belonging networks. (continued)**

| Label | Subregion name | region |  | Network assignment |
| --- | --- | --- | --- | --- |
| 201 | V5/MT+ | LOcC_L_4_2 |  | Dorsal Attention_L |
| 202 | V5/MT+ | LOcC_R_4_2 |  | Visual_R |
| 203 | OPC | LOcC_L_4_3 |  | Visual_L |
| 204 | OPC | LOcC_R_4_3 |  | Visual_R |
| 205 | iOccG | LOcC_L_4_4 |  | Visual_L |
| 206 | iOccG | LOcC_R_4_4 |  | Visual_R |
| 207 | msOccG | LOcC_L_2_1 |  | Visual_L |
| 208 | msOccG | LOcC_R_2_1 |  | Visual_R |
| 209 | lsOccG | LOcC_L_2_2 |  | Visual_L |
| 210 | lsOccG | LOcC_R_2_2 |  | Visual_R |
| 211 | mAmyg | Amyg_L_2_1 |  | subcortical_L |
| 212 | mAmyg | Amyg_R_2_1 |  | subcortical_R |
| 213 | lAmyg | Amyg_L_2_2 |  | subcortical_L |
| 214 | lAmyg | Amyg_R_2_2 |  | subcortical_R |
| 215 | rHipp | Hipp_L_2_1 |  | subcortical_L |
| 216 | rHipp | Hipp_R_2_1 |  | subcortical_R |
| 217 | cHipp | Hipp_L_2_2 |  | subcortical_L |
| 218 | cHipp | Hipp_R_2_2 |  | subcortical_R |
| 219 | vCa | BG_L_6_1 |  | subcortical_L |
| 220 | vCa | BG_R_6_1 |  | subcortical_R |
| 221 | GP | BG_L_6_2 |  | subcortical_L |
| 222 | GP | BG_R_6_2 |  | subcortical_R |
| 223 | NAC | BG_L_6_3 |  | subcortical_L |
| 224 | NAC | BG_R_6_3 |  | subcortical_R |
| 225 | vmPu | BG_L_6_4 |  | subcortical_L |
| 226 | vmPu | BG_R_6_4 |  | subcortical_R |
| 227 | dCa | BG_L_6_5 |  | subcortical_L |
| 228 | dCa | BG_R_6_5 |  | subcortical_R |
| 229 | dlPu | BG_L_6_6 |  | subcortical_L |
| 230 | dlPu | BG_R_6_6 |  | subcortical_R |
| 231 | mPFtha | Tha_L_8_1 |  | subcortical_L |
| 232 | mPFtha | Tha_R_8_1 |  | subcortical_R |
| 233 | mPMtha | Tha_L_8_2 |  | subcortical_L |
| 234 | mPMtha | Tha_R_8_2 |  | subcortical_R |
| 235 | Stha | Tha_L_8_3 |  | subcortical_L |
| 236 | Stha | Tha_R_8_3 |  | subcortical_R |
| 237 | rTtha | Tha_L_8_4 |  | subcortical_L |
| 238 | rTtha | Tha_R_8_4 |  | subcortical_R |
| 239 | PPtha | Tha_L_8_5 |  | subcortical_L |
| 240 | PPtha | Tha_R_8_5 |  | subcortical_R |

**Supplementary Table 3: All nodes and their belonging networks. (continued)**

| Label | Subregion name | region |  | Network assignment |
| --- | --- | --- | --- | --- |
| 241 | Otha | Tha_L_8_6 |  | subcortical_L |
| 242 | Otha | Tha_R_8_6 |  | subcortical_R |
| 243 | cTtha | Tha_L_8_7 |  | subcortical_L |
| 244 | cTtha | Tha_R_8_7 |  | subcortical_R |
| 245 | lPFtha | Tha_L_8_8 |  | subcortical_L |
| 246 | lPFtha | Tha_R_8_8 |  | subcortical_R |

SFG, Superior frontal gyrus;MFG, Middle frontal gyrus;IFG,Inferior frontal gyrus;OrG, orbital gyrus;PrG, Precentral gyrus;PCL, paracentral lobule;STG, Superior temporal gyrus;MTG, Middle temporal gyrus;ITG, Inferior temporal gyrus;FuG, fusiform gyrus;PhG, Parahippocampal gyrus;pSTS, Posterior superior temporal sulcus;SPL, superior parietal gyrus;IPL, Inferior parietal gyrus;PCun, Precuneus gyrus;PoG, Postcentral gyrus;INS, insula;CG,cingulate gyrus;MVOcC, medioventral occipital cortex;LOcC,lateral occipital cortex;Amyg, amygdala;Hipp, hippocampu; BG, Basal ganglia;Tha, thalamu

**Supplementary** **Figure 1**


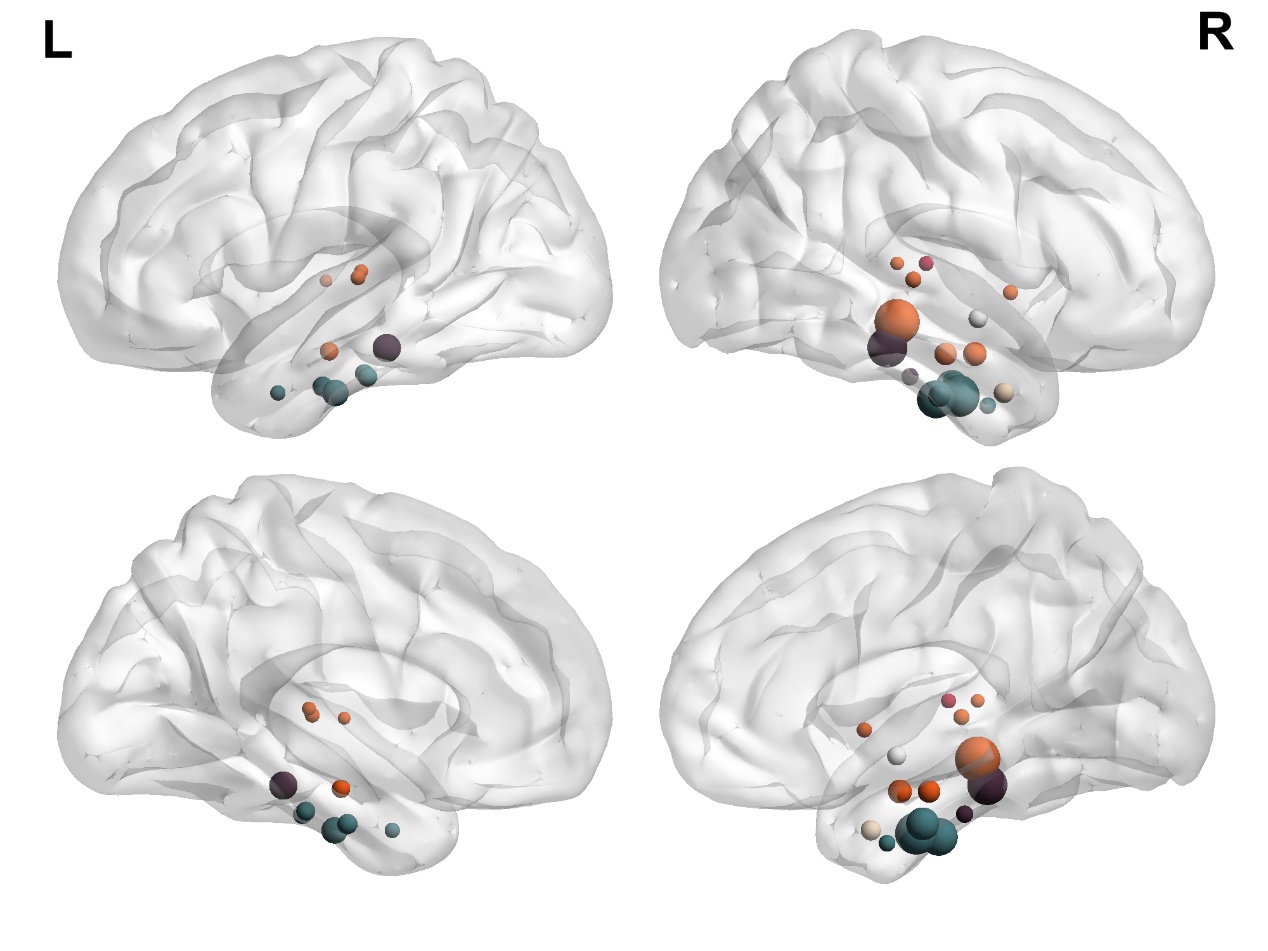


**Supplementary Figure 1**. Clustering analysis was conducted to examine the differential FC between the group of patients with TLE and HC groups, and use different colors to mark the network module where the node is located. Orange: Subcortical network; Purple: Visual network; Green: Limbic network; Light pink: Default mode network; White: Frontoparietal network. L, Left cerebral hemisphere; R, right cerebral hemisphere
